# What Is the Reality of Hiatal Hernia Management?—A Registry Analysis

**DOI:** 10.3389/fsurg.2020.584196

**Published:** 2020-10-22

**Authors:** Ferdinand Köckerling, Konstantinos Zarras, Daniela Adolf, Barbara Kraft, Dietmar Jacob, Dirk Weyhe, Christine Schug-Pass

**Affiliations:** ^1^Department of Surgery and Center for Minimally Invasive Surgery, Academic Teaching Hospital of Charité Medical School, Vivantes Hospital, Berlin, Germany; ^2^Department of Visceral, Minimally Invasive and Oncological Surgery, Marien Hospital Düsseldorf, Düsseldorf, Germany; ^3^StatConsult GmbH, Magdeburg, Germany; ^4^Department of General and Visceral Surgery, Diakonie Hospital, Stuttgart, Germany; ^5^Chirurgisch-Orthopädischer PraxisVerbund (COPV)—Hernia Center, Berlin, Germany; ^6^Department of General and Visceral Surgery, University Hospital of Visceral Surgery, Pius Hospital Oldenburg, Oldenburg, Germany

**Keywords:** hiatal hernia, nissen fundoplication, Toupet fundoplication, mesh, complications, recurrence

## Abstract

**Introduction:** To date, the guidelines for surgical repair of hiatal hernias do not contain any clear recommendations on the hiatoplasty technique with regard to the use of a mesh or to the type of fundoplication (Nissen vs. Toupet). This present 10-years analysis of data from the Herniamed Registry aims to investigate these questions.

**Methods:** Data on 17,328 elective hiatal hernia repairs were entered into the Herniamed Registry between 01.01.2010 and 31.12.2019. 96.4% of all repairs were completed by laparoscopic technique. One-year follow-up was available for 11,280 of 13,859 (81.4%) patients operated during the years 2010–2018. The explorative Fisher's exact test was used for statistical calculation of significant differences with an alpha = 5%. Since the annual number of cases in the Herniamed Registry in the years 2010–2012 was still relatively low, to identify significant differences the years 2013 and 2019 were compared.

**Results:** The use of mesh hiatoplasty for axial and recurrent hiatal hernias remained stable over the years from 2013 to 2019 at 20 and 45%, respectively. In the same period the use of mesh hiatoplasty for paraesophageal hiatal hernia slightly, but significantly, increased from 33.0 to 38.9%. The proportion of Nissen and Toupet fundoplications for axial hiatal hernia repair dropped from 90.2% in 2013 to 74.0% in 2019 in favor of “other techniques” at 20.9%. For the paraesophageal hiatal hernias (types II–IV) the proportion of Nissen and Toupet fundoplications was 68.1% in 2013 and 66.0% in 2019. The paraesophageal hiatal hernia repairs included a proportion of gastropexy procedures of 21.7% in 2013 and 18.7% in 2019. The recurrent hiatal hernia repairs also included a proportion of gastropexies 12.8% in 2013 and 15.1% in 2019, Nissen and Toupet fundoplications of 72.7 and 62.7%, respectively, and “other techniques” of 14.5 and 22.2%, respectively. No changes were seen in the postoperative complication and recurrence rates.

**Conclusion:** Clear trends are seen in hiatal hernia repair. The use of meshes has only slightly increased in paraesophageal hiatal hernia repairs. The use of alternative techniques has resulted in a reduction in the use of the “classic” Nissen and Toupet fundoplication surgical techniques.

## Introduction

According to the guidelines of the Society of American Gastrointestinal and Endoscopic Surgeons (SAGES), laparoscopic hiatal hernia repair is as effective as open transabdominal repair and is the preferred approach for the majority of hiatal hernias (1). The SAGES guidelines are specific to each type of hiatal hernia because the indications and treatment differ between the axial (type I) and paraesophageal hernias (PEH) (types II, III, and IV) (1). “The major clinical significance of a type I hiatal hernia is its association with gastro- esophageal reflux disease (GERD)” (1). According to the European Association of Endoscopic Surgery (EAES) guidelines for the management of GERD, “patients with continuously reduced quality of life, persistent troublesome symptoms and progression of disease despite adequate proton pump inhibitor therapy in dosage and intake should be offered laparoscopic antireflux surgery” (2). All symptomatic paraesophageal hiatal hernias (types II–IV) should be repaired (1). Recurrent hiatal hernia repair can safely be undertaken laparoscopically (1).

For management of hiatal hernias two technical aspects that potentially affect the outcome are still debated. These relate first to mesh-augmented hiatoplasty (1–11) and, second, to 360° Nissen fundoplication vs. 270° Toupet fundoplication (1–3, 12–14). So far, the guidelines to not contain any clear recommendations on either of these two surgical techniques.

As an alternative to the Nissen and Toupet fundoplication, there are reports in the literature of similar results being obtained with the DOR anterior hemifundoplication (15). In recent years the LINX system for magnetic esophageal sphincter augmentation and the EndoStim system for electrical stimulation of the lower esophageal sphincter have been added to the armamentarium for repair of axial hiatal hernia with GERD (16, 17).

For paraesophageal hernias with displacement of portions of the stomach far into the mediastinum (types III, IV) comparative studies have demonstrated that the quality of life with and without fundoplication is similar (18). Hence, fundophrenicopexy could be an alternative to fundoplication in the more severe paraesophageal hiatal hernias with no symptoms of reflux.

There is a paucity of studies containing recommendations for treatment of recurrences after hiatal hernia repair (19–22).

Against that background, the repair of hiatal hernias over the past 10 years was analyzed on the basis of data from the Herniamed Registry (23–26). The surgical techniques and outcomes were analyzed separately for axial (type I), paraesophageal (types II–IV), and recurrent hiatal hernias.

## Methods

“The Herniamed quality assurance study is a multicenter internet-based hernia registry with voluntary participating institutions which incorporate prospective data of patients who have undergone routine hernia surgery” (26). “These data are obtained from voluntarily participating hospitals and surgeons in Germany, Austria, and Switzerland” (26). “As part of the informed consent declaration, information provided to patients regarding participation in the Herniamed registry included the request that the hospital or medical practice providing treatment would like to be informed about any problem occurring after the operation and that patients have the opportunity to attend clinical examination” (20). “At 1-year follow-up, postoperative complications are once again reviewed when the general practitioner and the patient are asked to report any occurrences, pain at rest, pain on exertion, and chronic pain requiring treatment” (26). The publication by Lazar et al. (27) has provided impressive evidence of the role of patient-reported outcomes for recurrence and clinical symptoms following hiatal hernia repair.

In this retrospective analysis of prospective data entered into the Herniamed Registry the operative techniques and treatment results for axial (type I), paraesophageal (types II–IV), and recurrent hiatal hernias are presented separately and compared with each other. For analysis of hiatoplasty based on the Herniamed Registry data, a distinction can be made between the hiatoplasty techniques with suture only, with suture and mesh and mesh only. For fundoplication a distinction can be made between Nissen and Toupet fundoplication. Fundophrenicopexy can also be documented. Less frequently performed and new operative techniques can only be presented in the Herniamed Registry as “other techniques” while entering the name of the procedure into a free text field.

The surgical techniques and outcomes were calculated separately for each year from 2010 to 2019 and depicted as a curve to identify trends. Accordingly, 1-year follow-up results are available only for the years 2010–2018.

The explorative Fisher's exact test was used for statistical calculation of significant differences with an alpha = 5%. Since the annual number of cases in the Herniamed Registry in the years 2010–2012 was still relatively low and there was thus considerable fluctuation in the analysis results, the years 2013 and 2019 exhibiting a more stable trend were compared. That applies for comparison of the surgical techniques used and of the postoperative outcomes. Since only the results for the years 2010–2018 were available for 1-year follow-up, testing for significant differences in the recurrence rates is based on comparison of the results obtained for the years 2013 and 2018.

## Results

**Figure 1:**
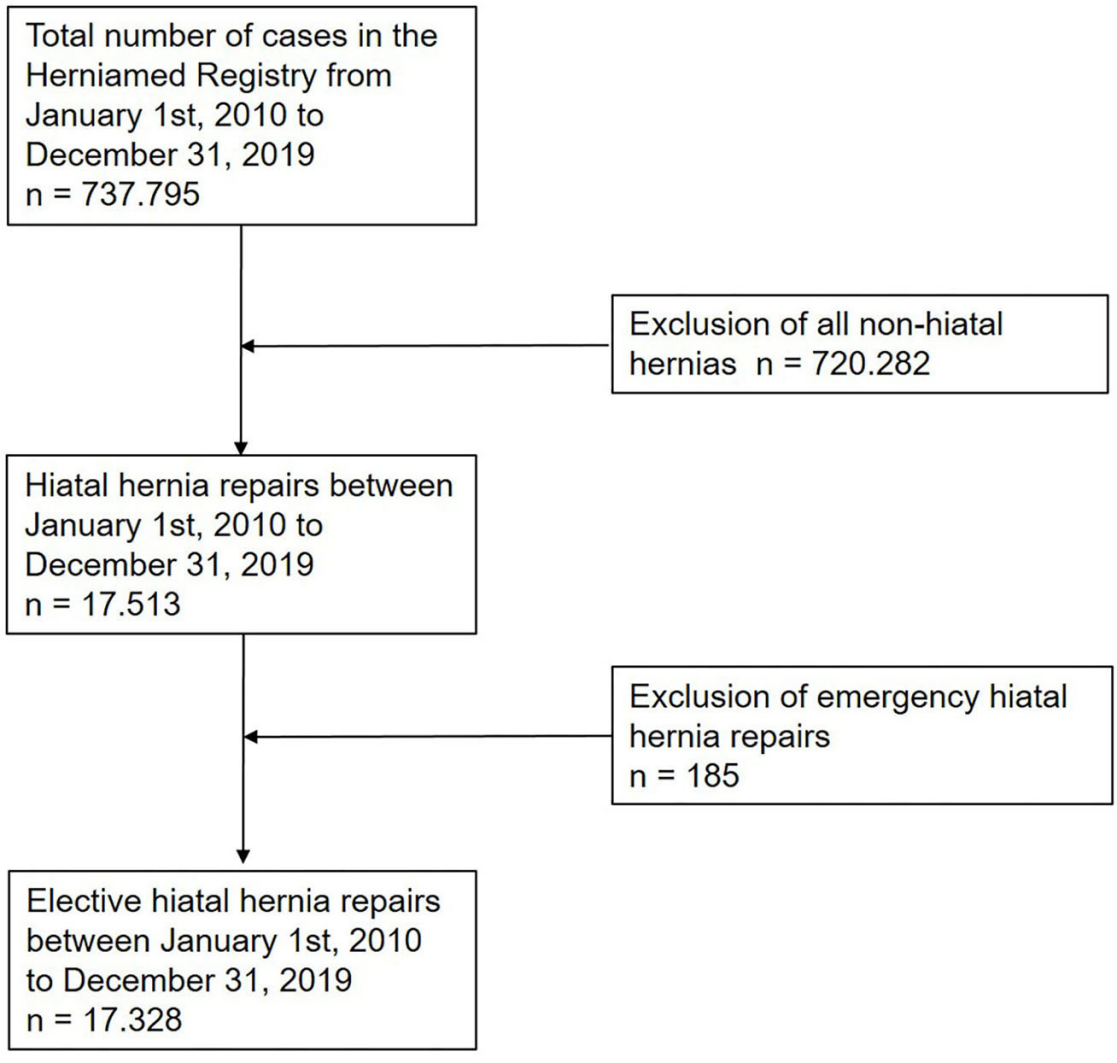
Flowchart of patient inclusion.

In total 17,328 patients with hiatal hernia repair were enrolled in the Herniamed Registry between 2010 and 2019 (Figure 1, Table 1). The proportion of open repairs, including conversions, was only 3.6%. 96.4% of all repairs were completed by laparoscopic technique. The increase in the number of cases each year was due to the rising number of hospitals and clinics participating in the Herniamed Registry. One-year follow-up was available for 11,280 of 13,859 (81.4%) of all patients for the years 2010–2018 (Table 1).

**Table 1:** Total case numbers per year with hiatal hernia repair.

**Year**	***n***	
2010	198	
2011	380	
2012	760	
2013	1,207	
2014	1,536	
2015	1,746	
2016	2,108	
2017	2,801	
2018	3,123	
2019	3,469	
Total	17,328	
1-year follow-up 2010–2018	*n* = 11,280/13,859	81,4 %

Of the 17,328 patients, 10,124 (58.4%) had axial (type I), 5,462 (31.5%) paraesophageal (types II-IV), and 1,742 (10.1%) recurrent hiatal hernia (Table 2). Nissen fundoplication was performed for a total of 6,054 (34.9%) cases, Toupet fundoplication for 6,911 (39.9%), and fundophrenicopexy for 1,706 (9.8%) patients (Table 2). Summarized as “other techniques” were 450 cases with implantation of a LINX magnetic esophageal sphincter augmentation device, 450 cases with hiatoplasty only with and without mesh, 319 anterior hemifundoplication (DOR), 158 reconstructions of the angle of His and 49 implantations of an EndoStim device.

**Table 2:** Distribution of hiatal hernia types and surgical procedures.

**Procedure**	**Axial (type I)**	**Paraesophageal (type II-IV)**	**Recurrent**	**Total**
Nissen fundoplication	3.632 (35.9 %)	1.831 (33.5 %)	591 (33.9 %)	6.054 (34.9 %)
Toupet fundoplication	4.489 (44.3 %)	1.859 (34.0 %)	563 (32.3 %)	6.911 (39.9 %)
Fundophrenicopexy	409 (4.0 %)	1.072 (19.6 %)	225 (12.9 %)	1.706 (9.8 %)
Other procedures	1.594 (15.8 %)	700 (12.9 %)	363 (20.9 %)	2657 (15.4 %)

**Figure 2:**
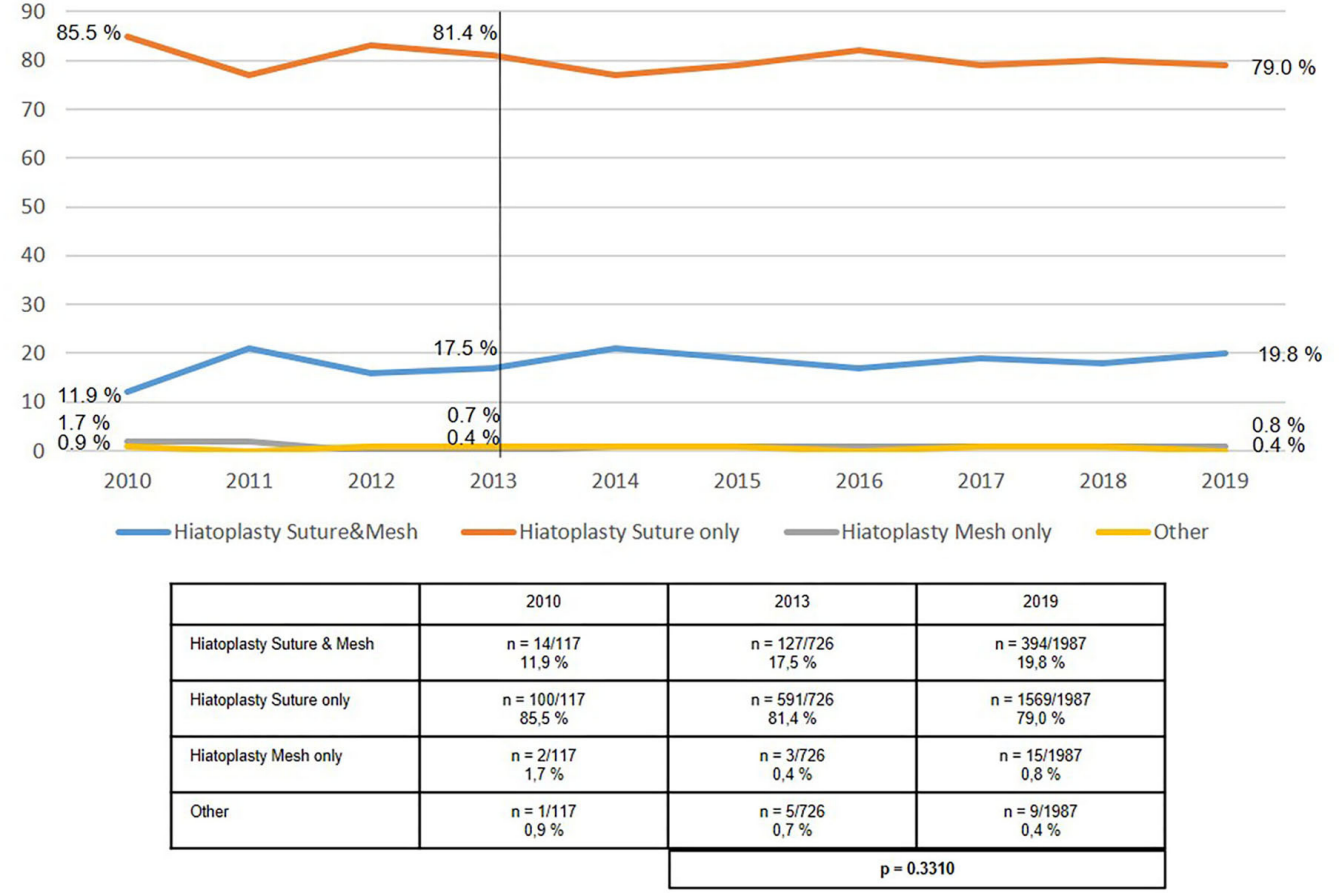
Elective primary repair of axial (type I) hiatal hernias—Technique of hiatoplasty 2010–2019.

**Figure 3:**
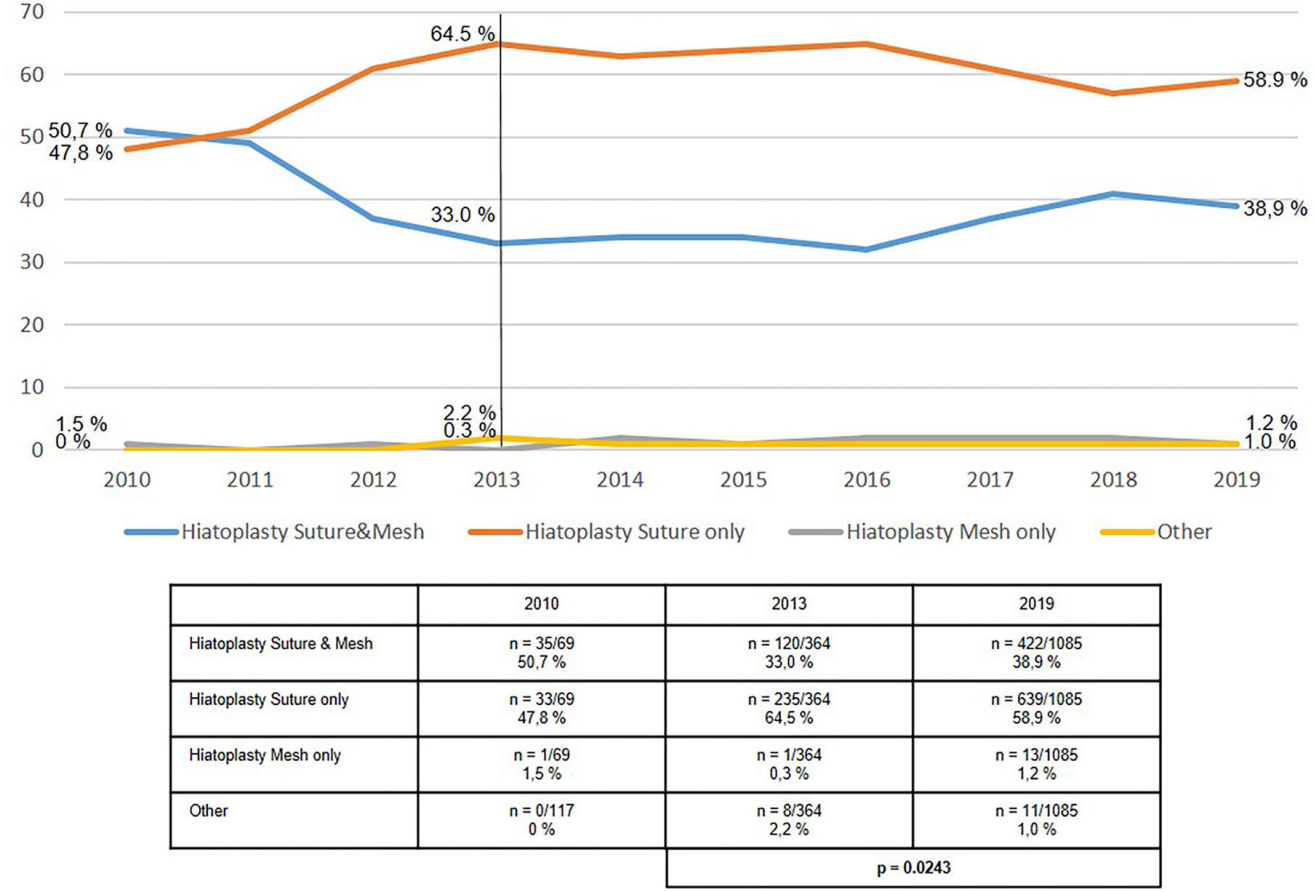
Elective primary repair of paraesophageal (type II-IV) hiatal hernias—Technique of hiatoplasty 2010–2019.

**Figure 4:**
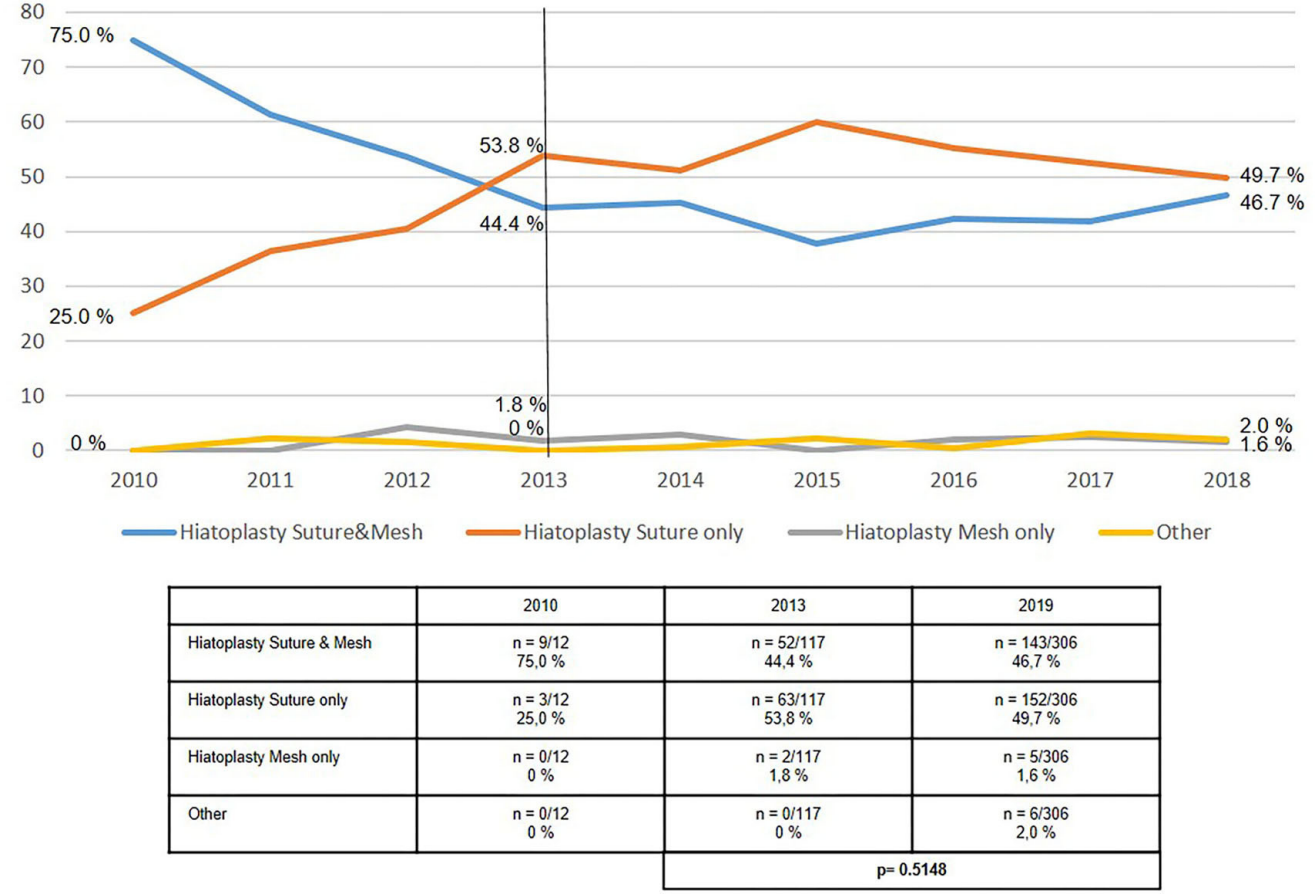
Elective recurrent hiatal hernia repairs—Technique of hiatoplasty 2010–2019.

The use of mesh hiatoplasty for axial hiatal hernias (type I), at around 20%, had not significantly changed over the period from 2013 to 2019 (Figure 2). The use of mesh hiatoplasty for paraesophageal hiatal hernia (types II–IV) increased slightly, but significantly, from 33.0 to 38.9% (Figure 3). No increase was seen in the use of mesh hiatoplasty for recurrent hiatal hernia at 44.4% in 2013 and 46.7% in 2019 (Figure 4). Hence, overall there was only a negligible increase in mesh hiatoplasty for hiatal hernia repair.

**Figure 5:**
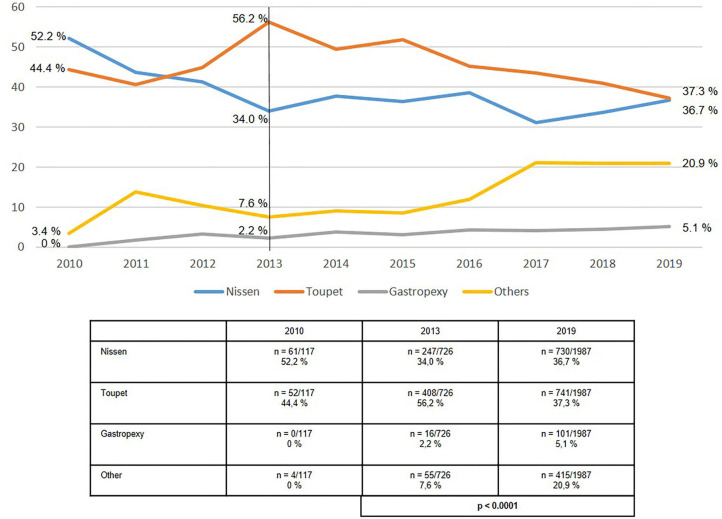
Elective primary repair of axial (type I) hiatal hernias—Surgical procedures 2010–2019.

The use of the “classic” antireflux operations, Nissen or Toupet fundoplication highly significantly declined between 2013 and 2019 in axial hiatal hernia repair (Figure 5). Whereas, in 2013 it accounted for more than 90% of procedures, its proportion in 2019 was only 74%. During the same period the proportion of “other techniques” increased from 7.6 to 20.9% and the proportion of fundophrenicopexy from 2.2 to 5.1%.

**Figure 6:**
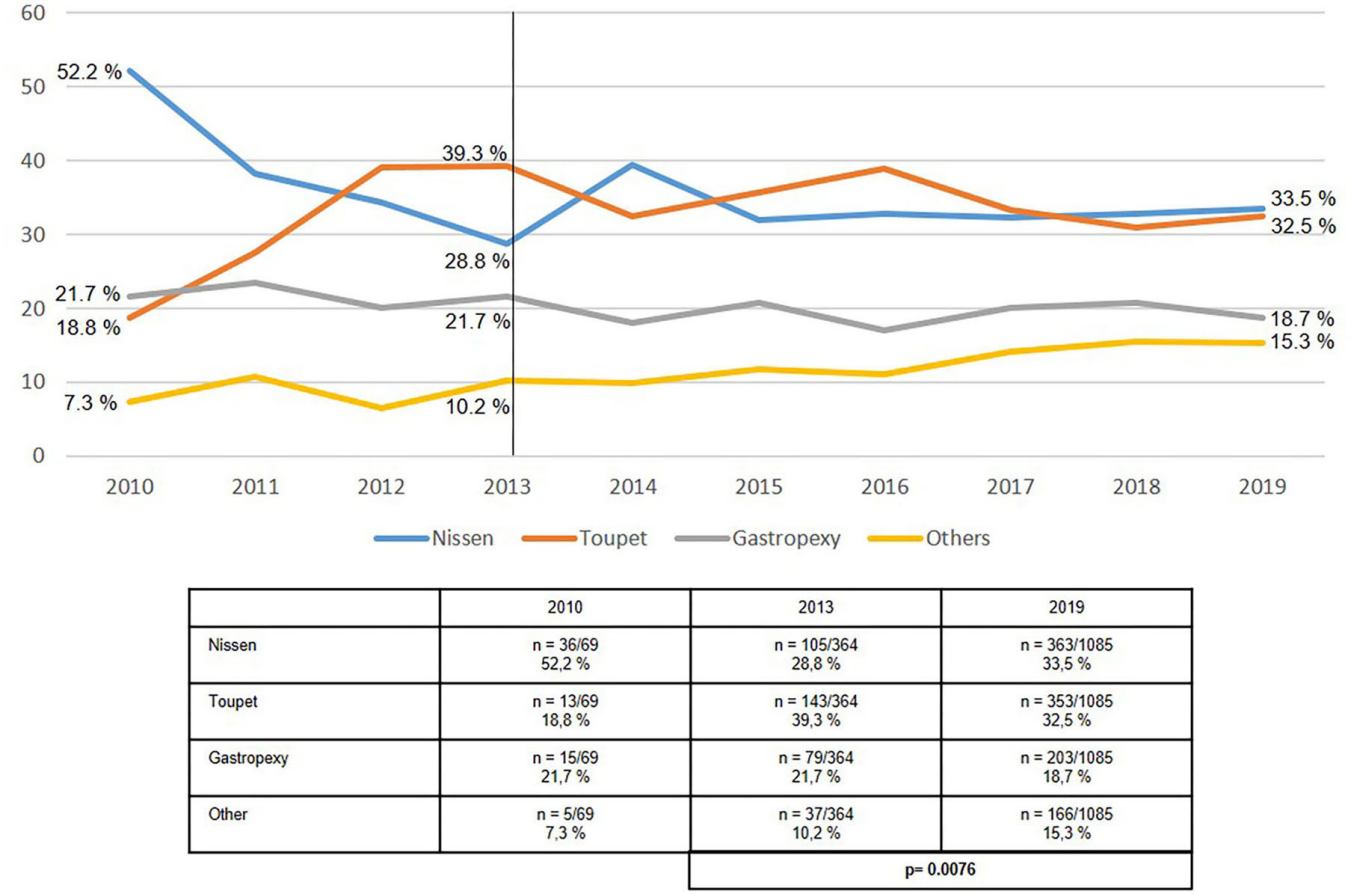
Elective primary repair of paraesophageal (types II-IV) hiatal hernias—Surgical procedures 2010–2019.

In the case of paraesophageal hiatal hernia repair, no change was seen in the proportion of Nissen and Toupet fundoplication procedures at 68.1% in 2013 and 66.0% in 2019 (Figure 6). Fundophrenicopexy remained stable over the observational period with a proportion of 21.7% in 2013 and 18.7% in 2019. A relevant increase was seen in “other techniques” from 10.2% in 2013 to 15.3% in 2019.

**Figure 7:**
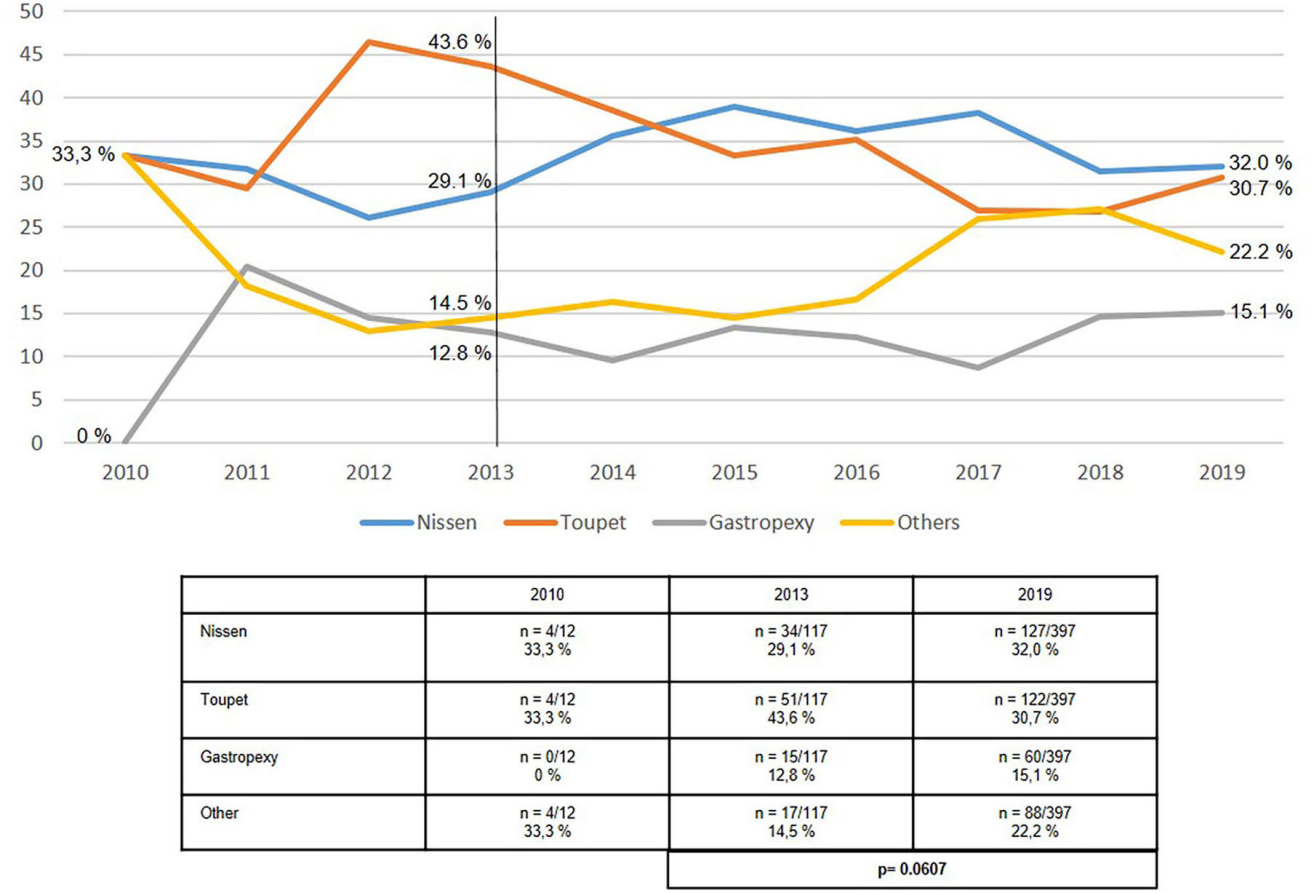
Elective recurrent hiatal hernia repairs—Surgical procedures 2010–2019.

The proportion of both Nissen and Toupet fundoplications used in recurrent hiatal hernia repair remained stable at around 60–70% (Figure 7). The proportion of fundophrenicopexy procedures in recurrent repair rose from 12.8% in 2013 to 15.1% in 2019. The proportion of “other techniques” increased from 14.5% in 2013 to 22.2% in 2019.

**Figure 8:**
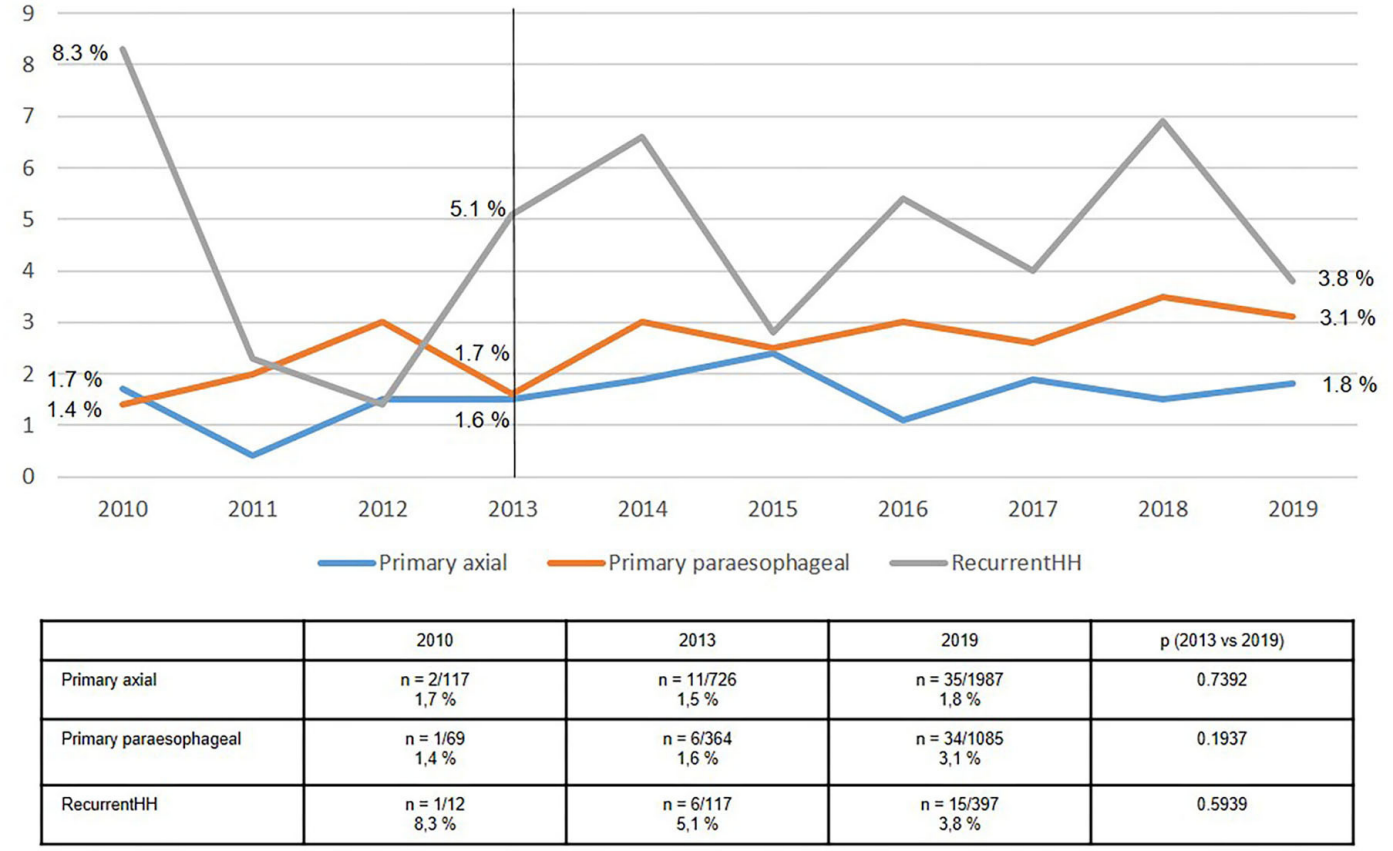
Postoperative complications of elective primary and recurrent hiatal hernia repairs 2010–2019.

As expected, the postoperative complication rate (Figure 8) was lowest for axial (type I) hiatal hernias at 1.8%, moderate for paraesophageal (types II–IV) hernias at 3.1%, and highest for recurrent hiatal hernias at 3.8% in 2019. There was no improvement in outcomes over the period from 2013 to 2019.

**Figure 9:**
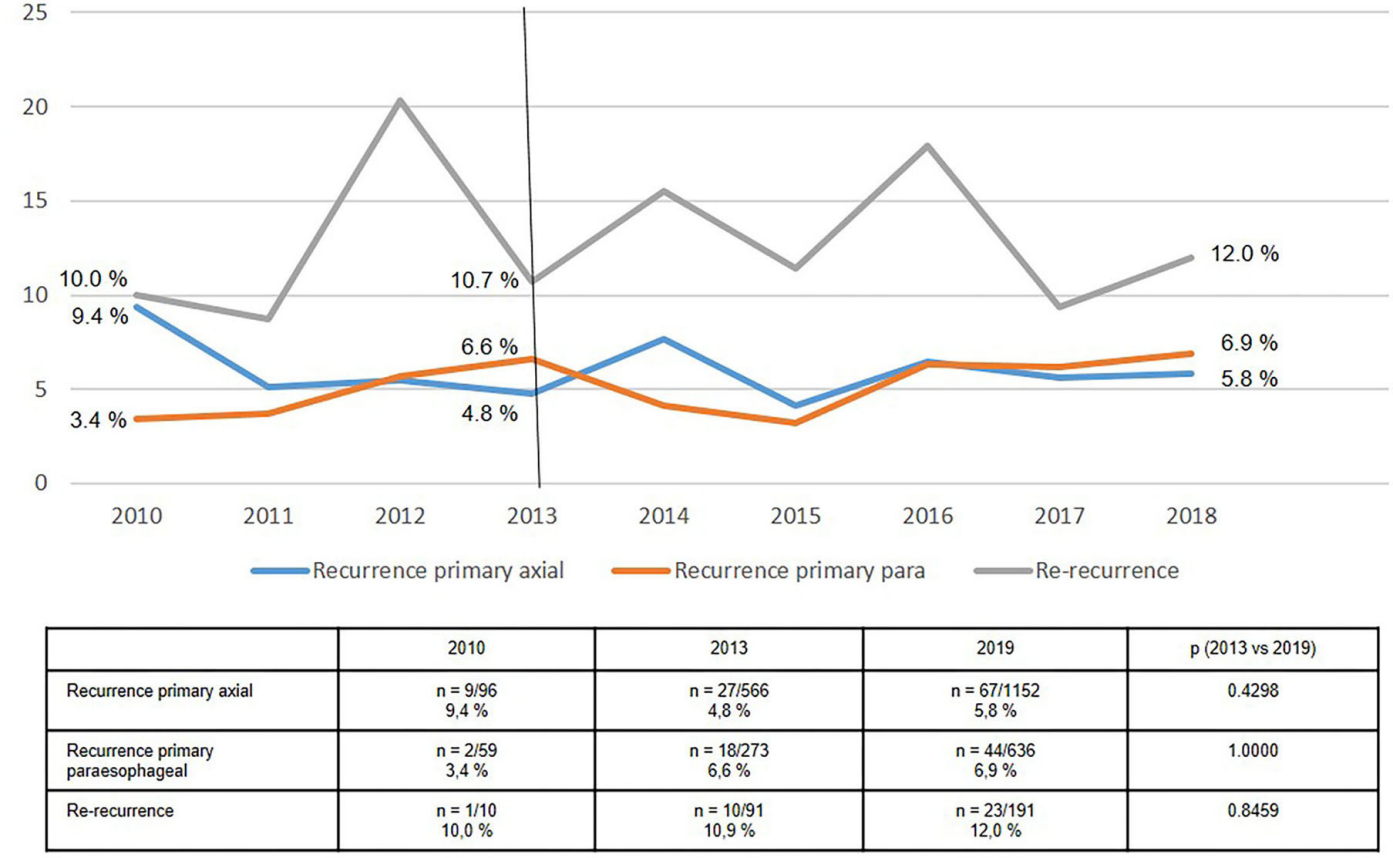
Recurrence rates of hiatal hernia repairs in 1-year follow-up 2010–2018.

Likewise, the recurrence rates for axial (type I) and paraesophageal (types II-IV) hiatal hernias were relatively stable at 5–6% (Figure 9). The re-recurrence rates following recurrent hiatal hernia repair fluctuated sharply, with average rates of around 10%. As such, the re-recurrence rates after recurrent hiatal hernia repair was almost twice that seen after axial and paraesophageal hiatal hernia repair.

## Discussion

This analysis reveals important trends in hiatal hernia repair.

The use of mesh hiatoplasty increased slightly, but significantly, only for paraesophageal hiatal hernia repair. For axial hiatal hernia as well as recurrent hiatal hernia the proportion of mesh hiatoplasty repairs remained stable between 2013 and 2019 at 20 and 45%, respectively.

Apparently surgeons had reservations about meshes due to the absence of clear recommendations in the guidelines (1, 2) for the use of meshes in hiatoplasty and because of the increasing number of publications about, albeit rare but severe, mesh complications (28). Furthermore, a meta-analysis investigating the use of a mesh for large hiatal hernias contained only five randomized controlled trials (RCTs) with 478 patients (29). That provided no clear evidence to support the role of mesh in hiatoplasty (29). Similarly, as regards the use of a mesh in recurrent hiatal hernia repair, there are no reports in the literature on the impact of a mesh in redo hiatoplasty. Hence, the absence of scientific evidence has led to surgeons adopting a somewhat more cautious stance with regard to the use of a mesh. Likewise, an analysis of data from the national database of the American College of Surgeons National Surgical Quality Improvement Program showed that a mesh was used only for a proportion of 38.1% paraesophageal hiatal hernia repairs (30). Here, the proportion of mesh hiatoplasty procedures in paraesophageal hiatal hernia repair declined from 46.2% in 2010 to 35.2% in 2017 (30). The reduction in mesh hiatoplasty did not result in less favorable outcomes (30). Magnetic resonance imaging of visible meshes can be used in future to demonstrate mesh shrinkage or potential complications (31).

For axial (type I) hiatal hernia the proportion of mesh hiatoplasty repairs since 2011 has only been 20%. Accordingly, more RCTs are urgently needed to investigate the influence of mesh hiatoplasty and mesh type in axial, paraesophageal and recurrent hiatal hernia repair. Similarly, a multivariable analysis of registry data did not find any evidence that mesh hiatoplasty had a favorable influence on the recurrence rate following axial and paraesophageal hiatal hernia repair (23). As such, the cautious approach taken by surgeons to the use of a mesh in hiatoplasty is understandable.

However, the decline in the percentage proportion of the “classic” antireflux repair procedures, Nissen and Toupet fundoplication, in axial (type I) and paraesophageal (types II–IV) hiatal hernia repair is surprising. On the other hand, the “other techniques” (LINX magnetic esophageal sphincter augmentation, hiatoplasty with or without mesh, anterior DOR hemifundoplication, reconstruction of the angle of His and EndoStim device implantation) increased for both axial (type I) and paraesophageal (types II-IV) hiatal hernias from 7.6 to 20.9% and from 10.2 to 15.3%, respectively. To date, none of these techniques has been recommended in the guidelines due to a lack of evidence (1, 2). Therefore, the increasing use of these “other techniques” in hiatal hernia repair must be viewed in a critical light.

Gastropexy, in addition to Nissen and Toupet fundoplication, has become an established procedure for paraesophageal as well as for recurrent hiatal hernias at 15–20%. Good results have been reported for anterior gastropexy in paraesophageal hiatal hernia repair (32). Gastropexy lends itself, in particular, for large paraesophageal hiatal hernias without reflux symptoms and for recurrences where redo fundoplication is technically contraindicated.

Depending on the technical level of difficulties faced, the postoperative complication rate after axial repair was 2%, after paraesophageal around 3% and after recurrent hiatal hernia repair it fluctuated between 3.8 and 5.1%. Overall, there was no decline in the postoperative complication rate following hiatal hernia repair. These findings concord with those from the Danish nationwide health registry (21).

The recurrence rate at 1-year follow-up for both axial and paraesophageal hiatal hernia over the period from 2013 to 2018 remained stable at around 5–6%. Conversely, the re-recurrence rate following recurrent hiatal hernia repair was above 10%. In the registry the diagnostic procedure for the recurrence is not documented.

The recurrence rates following hiatal hernia repair reported in the literature are between 8.0 and 26% (4), with differences seen depending on the type of hiatal hernia, hiatoplasty technique, antireflux operation and follow-up duration. Accordingly, an increase in the recurrence rate to as high as the reference range must be expected if this patient group is followed up for a longer period.

This analysis is subject to the usual limitations of a registry study. A contract was made with every participating institution in which the responsible surgeon committed to ensuring complete and correct data entry. At the time of certification of hernia centers the auditor can conduct spot checks of the Herniamed documentation. Furthermore, a relevant proportion of patients was lost to follow-up. The results presented here were generated by an evaluation tool for the years under review. It was not possible to take account of differences in patient characteristics.

In summary, this analysis shows a stable proportion of mesh hiatoplasty procedures in axial at 20% and in recurrent hiatal hernia repair at 45%, but a slight increase in paraesophageal hiatal hernia repair. Nissen or Toupet fundoplication was used less frequently in axial and paraesophageal hiatal hernia repair. On the other hand, “other techniques,” such as the LINX and EndoStim devices, hiatoplasty only, anterior DOR hemifundoplication, and reconstruction of the angle of His, were used more often. Gastropexy was consistently used in about 20% of paraesophageal hiatal hernia repairs. To date, this has not resulted in any changes in either the postoperative complication rates or the recurrence rate.

## Data Availability Statement

All datasets presented in this study are included in the article/supplementary material.

## Ethics Statement

Ethical review and approval was not required for the study on human participants in accordance with the local legislation and institutional requirements. The patients/participants provided their written informed consent to participate in this study.

## Author Contributions

FK study concept, literature review, manuscript writing, revision of manuscript, and final approval of the manuscript. KZ, BK, DJ, DW, and CS-P study concept, literature review, manuscript revision, and final approval of the manuscript. DA study concept, statistical analysis, manuscript revision, and final approval of the manuscript. All authors contributed to the article and approved the submitted version.

## Conflict of Interest

FK reports grants to fund Herniamed from Johnson & Johnson, Norderstedt, grants from Karl Storz, Tuttlingen, grants from pfm medical, Cologne, grants from Dahlhausen, Cologne, grants from B Braun, Tuttlingen, grants from MenkeMed, Munich, grants from Bard, Karlsruhe, during the conduct of the study; personal fees from Bard, Karlsruhe, outside the submitted work. DA was employed by the company StatConsult GmbH. The remaining authors declare that the research was conducted in the absence of any commercial or financial relationships that could be constructed as a potential conflict of interest. The reviewer AS declared a past co-authorship with one of the authors FK to the handling editor.

## References

[1] Kohn GP Price RR deMeester SR Zehetner J Muensterer OJ Awad Z. Guidelines for the management of hiatal hernia. Surg Endosc. (2013) 27:4409–28. 10.1007/s00464-013-3173-3 24018762

[2] Fuchs KH Babic B Breithaupt W Dallemagne B Fingerhut A Furnee E. EAES recommendations for the management of gastroesophageal reflux disease. Surg Endosc. (2014) 28:1753–73. 10.1007/s00464-014-3431-z 24789125

[3] Seo HS Choi M Son SY Kim MG Han DS Lee HH. Evidence-based practice guideline for surgical treatment of gastroesophageal reflux disease 2018. J Gastric Cancer. (2018) 18:313–27. 10.5230/jgc.2018.18.e41 30607295 PMC6310769

[4] Antoniou SA Antoniou GA Koch OO Pointner R Granderath FA. Lower recurrence rates after mesh-reinforced versus simple hiatal hernia repair: a meta-analysis of randomized trials. Surg Laparosc Endosc Percutan Tech. (2012) 22:498–502. 10.1097/SLE.0b013e3182747ac2 23238375

[5] Müller-Stich BP Kenngott HG Gondan M Stock C Linke GR Fritz F. Use of mesh in laparoscopic paraesophageal hernia repair: a meta-analysis and risk-benefit analysis. PLoS ONE. (2015) 10:e0139547. 10.1371/journal.pone.0139547 26469286 PMC4607492

[6] Furnée E Hazebroek E. Mesh in laparoscopic large hiatal hernia repair: a systematic review of the literature. Surg Endosc. (2013) 27:3998–4008. 10.1007/s00464-013-3036-y 23793804

[7] Tam V Winger DG Nason KS. A systematic review and meta-analysis of mesh vs suture cruroplasty in laparoscopic large hiatal hernia repair. Am J Surg. (2016) 211:226–38. 10.1016/j.amjsurg.2015.07.007 26520872 PMC5153660

[8] Antoniou SA Koch OO Antoniou GA Pointner R Granderath FA. Mesh-reinforced hiatal hernia repair: a review on the effect on postoperative dysphagia and recurrence langenbecks. Arch Surg. (2012) 397:19–27. 10.1007/s00423-011-0829-0 21792699

[9] Antoniou SA Müller-Stich BP Antoniou GA Köhler G Luketina RR Koch OO. Laparoscopic augmentation of the diaphragmatic hiatus with biologic mesh versus suture repair: a systematic review and meta-analysis langenbecks. Arch Surg. (2015) 400:577–83. 10.1007/s00423-015-1312-0 26049745

[10] Huddy JR Markar SR Ni MZ Morino M Targarona EM Zaninotto G. Laparoscopic repair of hiatus hernia: does mesh type influence outcome? A meta-analysis and European survey study. Surg Endosc. (2016) 30:5209–21. 10.1007/s00464-016-4900-3 27129568

[11] Memon MA Memon B Yunus RM Khan S. Suture cruroplasty versus prosthetic hiatal herniorrhaphy for large hiatal hernia: a meta-analysis and systematic review of randomized controlled trials. Ann Surg. (2016) 263:258–66. 10.1097/SLA.0000000000001267 26445468

[12] Tan G Yang Z Wang Z. Meta-analysis of laparoscopic total (Nissen) versus posterior (Toupet) fundoplication for gastro-oesophageal reflux disease based on randomized clinical trials. ANZ J Surg. (2011) 81:246–52. 10.1111/j.1445-2197.2010.05481.x 21418467

[13] Tian ZC Wang B Shan CX Zhang W Jiang DZ Qiu M. A meta-analysis of randomized controlled trials to compare long-term outcomes of nissen and toupet fundoplication for gastroesophageal reflux disease. PLoS One. (2015) 10:e0127627. 10.1371/journal.pone.0127627 26121646 PMC4484805

[14] Broeders JA Mauritz FA Ahmed Ali U Draaisma WA Ruurda JP Gooszen HG. Systematic review and meta-analysis of laparoscopic Nissen (posterior total) versus Toupet (posterior partial) fundoplication for gastro-oesophageal reflux disease. Br J Surg. (2010) 97:1318–30. 10.1002/bjs.7174 20641062

[15] Raue W Ordemann J Jacobi CA Menenakos C Buchholz A Hartmann J. Nissen versus dor fundoplication for treatment of gastroesophageal reflux disease: a blinded randomized clinical trial. Dig Surg. (2011) 28:80–6. 10.1159/000323630 21293136

[16] Skubleny D Switzer N Dang J Gill RS Shi X de Gara C. LINX® magnetic esophageal sphincter augmentation versus nissen fundoplication for gastroesophageal reflux disease: a systematic review and meta-analysis. Surg Endosc. (2017) 31:3078–84. 10.1007/s00464-016-5370-3 27981382

[17] Soffer E Rodríguez L Rodriguez P Gómez B Neto MG Crowell MD. Effect of electrical stimulation of the lower esophageal sphincter in gastroesophageal reflux disease patients refractory to proton pump inhibitors. World J Gastrointest Pharmacol Ther. (2016) 7:145–55. 10.4292/wjgpt.v7.i1.145 26855821 PMC4734948

[18] Svetanoff WJ Pallati P Nandipati K Lee T Mittal SK. Does the addition of fundoplication to repair the intra-thoracic stomach improve quality of life? Surg Endosc. (2016) 30:4590–7. 10.1007/s00464-016-4796-y 26905576

[19] Symons NR Purkayastha S Dillemans B Athanasiou T Hanna GB Darzi A. Laparoscopic revision of failed antireflux surgery: a systematic review. Am J Surg. (2011) 202:336–43. 10.1016/j.amjsurg.2011.03.006 21788005

[20] Furnée EJ Draaisma WA Broeders IA Gooszen HG. Surgical reintervention after failed antireflux surgery: a systematic review of the literature. J Gastrointest Surg. (2009) 13:1539–49. 10.1007/s11605-009-0873-z 19347410 PMC2710493

[21] Ljungdalh JS Rubin KH Durup J Houlind KC. Reoperation after antireflux surgery: a population-based cohort study. Br J Surg. (2020). 10.1002/bjs.11672. [Epub ahead of print]. 32484246

[22] van Beek DB Auyang ED Soper NJ. A comprehensive review of laparoscopic redo fundoplication. Surg Endosc. (2011) 25:706–12. 10.1007/s00464-010-1254-0 20661749

[23] Köckerling F Trommer Y Zarras K Adolf D Kraft B Weyhe D. What are the differences in the outcome of laparoscopic axial (I) versus paraesophageal (II-IV) hiatal hernia repair? Surg Endosc. (2017) 31:5327–41. 10.1007/s00464-017-5612-z 28597286 PMC5715051

[24] Stechemesser B Jacob DA Schug-Paß C Köckerling F. Herniamed: an internet-based registry for outcome research in hernia surgery. Hernia. (2012) 16:269–76. 10.1007/s10029-012-0908-3 22389073

[25] Kyle-Leinhase I Köckerling F Jørgensen LN Montgomery A Gillion JF Rodriguez JAP. Comparison of hernia registries: the CORE project. Hernia. (2018) 22:561–75. 10.1007/s10029-017-1724-6 29307057 PMC6061062

[26] Köckerling F Simon T Hukauf M Hellinger A Fortelny R Reinpold W. The importance of registries in the postmarketing surveillance of surgical meshes. Ann Surg. (2018) 268:1097–104. 10.1097/SLA.0000000000002326 28594740 PMC6250300

[27] Lazar DJ Birkett DH Brams DM Ford HA Williamson C Nepomnayshy D. Long-term patient-reported outcomes of paraesophageal hernia repair. JSLS. (2017) 21:e2017.00052. 10.4293/JSLS.2017.00052 29162971 PMC5683814

[28] Spiro C Quarmby N Gananadha S. Mesh-related complications in paraoesophageal repair: a systematic review. Surg Endosc. (2020). 10.1007/s00464-020-07723-0. [Epub ahead of print]. 32556700

[29] Memon MA Siddaiah-Subramanya M Yunus RM Memon B Khan S. Suture cruroplasty versus mesh hiatal herniorrhaphy for large hiatal hernias (HHs): an updated meta-analysis and systematic review of randomized controlled trials. Surg Laparosc Endosc Percutan Tech. (2019) 29:221–32. 10.1097/SLE.0000000000000655 30855402

[30] Schlosser KA Maloney SR Prasad T Augenstein VA Heniford BT Colavita PD. Mesh reinforcement of paraesophageal hernia repair: trends and outcomes from a national database. Surgery. (2019) 166:879–85. 10.1016/j.surg.2019.05.014 31288936

[31] Weyhe D Klinge U Uslar VN Tabriz N Kluge A. Follow up data of MRI-visible synthetic meshes for reinforcement in large hiatal hernia in comparison to none-mesh repair - a prospective cohort study. Front Surg. (2019) 6:17. 10.3389/fsurg.2019.00017 31058163 PMC6477929

[32] Daigle CR Funch-Jensen P Calatayud D Rask P Jacobsen B Grantcharov TP. Laparoscopic repair of paraesophageal hernia with anterior gastropexy: a multicenter study. Surg Endosc. (2015) 29:1856–6. 10.1007/s00464-014-3877-z 25294550

